# Fiducial Inference in Linear Mixed-Effects Models

**DOI:** 10.3390/e27020161

**Published:** 2025-02-03

**Authors:** Jie Yang, Xinmin Li, Hongwei Gao, Chenchen Zou

**Affiliations:** School of Mathematics and Statistics, Qingdao University, Qingdao 266071, Chinaxmli@qdu.edu.cn (X.L.); gaohongwei@qdu.edu.cn (H.G.)

**Keywords:** confidence interval, fiducial inference, LME, MCMC, zero-variance inference, 62F99

## Abstract

We develop a novel framework for fiducial inference in linear mixed-effects (LME) models, with the standard deviation of random effects reformulated as coefficients. The exact fiducial density is derived as the equilibrium measure of a reversible Markov chain over the parameter space. The density is equivalent in form to a Bayesian LME with noninformative prior, while the underlying fiducial structure adds new benefits to unify the inference of random effects and all other parameters in a neat and simultaneous way. Our fiducial LME needs no additional tests or statistics for zero variance and is more suitable for small sample sizes. In simulation and empirical analysis, our confidence intervals (CIs) are comparable to those based on Bayesian and likelihood profiling methods. And our inference for the variance of random effects has competitive power with the likelihood ratio test.

## 1. Introduction

The linear mixed-effects (LME) model is very popular in many fields like physics, biology and social sciences [1], where both within-subject and between-subject variations are studied. There have been a wealth of LME-based methodologies developed in the past decades, such as model selection [2], model averaging [3], semiparametric [4] and nonparametric [5] estimations, etc. Large-sample asymptotics of likelihood-based LME models are well studied [6]. Bayesian LME models also perform well under medium-to-large samples. For small samples, the inference based on the maximum likelihood needs finite-sample corrections or approximations [7], and Bayesian models rely much more on the prior.

The analysis of variance (ANOVA) and t-test summaries are two common ways of statistical inference in LME models. For fixed effects, *F* statistics in ANOVA and *t* statistics are available in most software. However, testing zero-variance components in random effects is challenging for small samples and has not been well resolved in the statistical literature. When some of the within-subject variances are zero or close-to-zero, Bayesian estimations may encounter issues [8]. And the inference of zero variance involves additionally constructed statistics like the profiling restricted maximum likelihood (REML) ratio test [9] or posterior evidence ratio with properly built prior [10]. Comparisons among multiple nested models are often unskippable.

From a frequentist viewpoint, aside from asymptotic approximations, the inference can be facilitated by nonparametric approaches [11,12], which are also known to be performant in LME models for small sample sizes. According to a comparative study [13] on various bootstrap methods in LME models, a nonparametric bootstrap with resampling on subjects is recommended [11].

Fiducial inference, initiated by Fisher [14] and revitalized in the 21st century [15], has unique advantages in small-sample inference in parametric statistical models, despite its limits in nonparametric statistics and computing cost [16]. Unlike Bayesian posterior built upon prior, fiducial distribution derived from data generation equations thus relieves the need for prior. For LME, Li et al. [17] proposed a fiducial test for within-subject variances, but they did not consider other parameters. Hari et al. [18] studied a two-component LME with partially known structures on covariances. Hannig et al. [19] developed a generalized fiducial inference (GFIlmm) for interval data. Although GFIlmm can be adapted to non-interval cases by artificially adding the intervals, the estimations vary with the interval widths. And its numerical algorithm in practice is inefficient on models with more than nine parameters.

In this study, we propose a new framework for non-asymptotic inference in LME models via a fiducial approach, which reduces computational burden and does not require duplicates within each group. Moreover, our approach naturally incorporates the zero-variance inference, thus cutting the labor of extra tests and calculation.

The rest of this article is organized as follows: Section 2 and Section 3 are about the methodology and algorithms. Section 4 is the simulation and Section 5 is a real data illustration. Section 6 is the conclusion and discussion of our study.

## 2. Fiducial Distribution in LME

We assume the data is generated as

(1) $$y_{i}=x_{i}\beta +z_{i}\gamma_{i}+e_{i},i=1,\cdots ,m,$$

where $\beta \in R^{p\times 1}$ is a vector of fixed coefficients; $\gamma_{i}∼N_{q}\left(0,D\right)$ is a random coefficient vector; $y_{i}$ is the $n_{i}\times 1$ response of the *i*th subject; $x_{i}$ and $z_{i}$ are the $n_{i}\times p$ and $n_{i}\times q$ covariate matrix of fixed and random effects, respectively; and $e_{i}∼N\left(0,\sigma^{2}I_{n_{i}}\right)$ is independent of $\gamma_{i}$, $n_{i}\geq 1$, i=1,…,m, $\sum_{i=1}^{m}n_{i}=n$. In this study, we assume a diagonal $D=diag(\sigma_{1}^{2},\ldots ,\sigma_{q}^{2})$.

### 2.1. Conditional Fiducial Distribution

Let $\delta ={\left(\sigma_{1},\ldots ,\sigma_{q}\right)}^{\prime }$, $U_{i}\overset{i.i.d}{∼}N\left(0,I_{q}\right)$ and $z_{U,i}=z_{i}diag\left(U_{i}\right)$, then model (1) can be rewritten as

$$y_{i}=x_{i}\beta +z_{U,i}\delta +e_{i},i=1,\cdots ,m.$$

It can be further specified in matrix form as

(2) $$Y=X\beta +Z_{U}\delta +\epsilon ,$$

with $Y={\left(y_{1}^{\prime },\cdots ,y_{m}^{\prime }\right)}^{\prime }$, $X={\left(x_{1}^{\prime },\cdots ,x_{m}^{\prime }\right)}^{\prime }$, $Z_{U}={\left(z_{U,1}^{\prime },\cdots ,z_{U,m}^{\prime }\right)}^{\prime }$, $U={\left(U_{1}^{\prime },\cdots ,U_{m}^{\prime }\right)}^{\prime }$ and $\epsilon ={\left(e_{1}^{\prime },\ldots ,e_{m}^{\prime }\right)}^{\prime }$. The $\sigma_{j}$ in random effects are formulated as a coefficient vector δ in Equation (2). This brings two benefits: 1. The inference of random effects can be realized by a general fiducial recipe [16] like all other parameters, avoiding extra tests and statistics like the LR tests. 2. We do not need $n_{i}\geq q$. The model can still be estimated even if $n_{i}=1$. So, our method can handle inadequate within-subject measures.

Let $\theta ={\left(\beta^{\prime },\delta^{\prime }\right)}^{\prime }$, $X_{U}={\left(X,Z_{U}\right)}_{n\times (p+q)}$ and $P_{U}=X_{U}{\left(X_{U}^{\prime }X_{U}\right)}^{-1}X_{U}^{\prime }$, then model (2) given U=u can be reformed as

$$Y=X\beta +Z_{u}\delta +\epsilon =X_{u}\theta +\epsilon ,$$

For observed Y=y, the conditional fiducial density of θ and $\sigma^{2}$ given *u*, denoted as $r_{y}\left(\theta |\sigma^{2},u\right)$ and $r_{y}\left(\sigma^{2}|u\right)$, respectively, could be derived from the conditional fiducial quantities [17] below:

(3) $$\begin{array}{cc} Q_{y}\left(\sigma^{2}|u\right) & =y^{\prime }\left(I-P_{u}\right)y/\xi , \end{array}$$

(4) $$\begin{array}{cc} Q_{y}\left(\theta |u\right) & ={\left(X_{u}^{\prime }X_{u}\right)}^{-1}X_{u}^{\prime }y+Q_{y}^{\frac{1}{2}}\left(\sigma^{2}|u\right){\left(X_{u}^{\prime }X_{u}\right)}^{-\frac{1}{2}}\zeta , \end{array}$$

where $\xi ∼\chi_{n-p-q}^{2}$, $\zeta ∼N(0,I_{p+q})$ independently, irrespective of any parameters in an LME model. Let $\eta ={\left(\theta^{\prime },\sigma^{2}\right)}^{\prime }$, then the conditional fiducial density of η given *u* is

(5) $$\begin{array}{cc} \; & r_{y}\left(\eta |u\right)=r_{y}\left(\sigma^{2}|u\right)r_{y}\left(\theta |\sigma^{2},u\right)\\ = & \frac{exp\left(-\frac{y^{\prime }\left(I-P_{u}\right)y}{2\sigma^{2}}\right){\left(\frac{y^{\prime }\left(I-P_{u}\right)y}{2\sigma^{2}}\right)}^{\frac{n-p-q}{2}-1}}{\sigma^{4}\Gamma \left(\frac{n-p-q}{2}\right)}\frac{exp\left(-\frac{1}{2\sigma^{2}}{\left(\theta -{\left(X_{u}^{\prime }X_{u}\right)}^{-1}X_{u}^{\prime }y\right)}^{\prime }\left(X_{u}^{\prime }X_{u}\right)\left(\theta -{\left(X_{u}^{\prime }X_{u}\right)}^{-1}X_{u}^{\prime }y\right)\right)}{{\left(2\pi \right)}^{\frac{p+q}{2}}\sigma^{p+q}{\left|X_{u}^{\prime }X_{u}\right|}^{-\frac{1}{2}}}\\ = & \frac{{\left(y^{\prime }\left(I-P_{u}\right)y\right)}^{\frac{n-p-q}{2}-1}}{\sigma^{n-p-q+2}\Gamma \left(\frac{n-p-q}{2}\right)2^{\frac{n-p-q}{2}-1}}\frac{exp(-\frac{∥y-X_{u}\theta {∥}^{2}}{2\sigma^{2}})}{{\left(2\pi \right)}^{\frac{p+q}{2}}\sigma^{p+q}{\left|X_{u}^{\prime }X_{u}\right|}^{-\frac{1}{2}}}\\ = & \frac{2|X_{u}^{\prime }X_{u}{|}^{\frac{1}{2}}{\left(y^{\prime }\left(I-P_{u}\right)y\right)}^{\frac{n-p-q}{2}-1}}{\Gamma \left(\frac{n-p-q}{2}\right)\pi^{\frac{p+q-n}{2}}}\frac{exp(-\frac{∥y-X_{u}\theta {∥}^{2}}{2\sigma^{2}})}{{\left(2\pi \right)}^{\frac{n}{2}}\sigma^{n+2}}\overset{▵}{=}C\left(X_{u},y\right)\frac{p(y|u,\eta )}{\sigma^{2}}. \end{array}$$

Here, $C\left(X_{u},y\right)=\frac{2|X_{u}^{\prime }X_{u}{|}^{\frac{1}{2}}{\left(y^{\prime }\left(I-P_{u}\right)y\right)}^{\frac{n-p-q}{2}-1}}{\Gamma \left(\frac{n-p-q}{2}\right)\pi^{(p+q-n)/2}}$ and p(y|u,η) is the likelihood function of (2) given U=u.

*U* is invisible in reality. But, it is easy to see that $U_{i}|\eta ,y∼N_{q}\left(E\left(U_{i}|\eta ,y\right),Var\left(U_{i}|\eta ,y\right)\right)$ with

(6) $$\begin{array}{cc} E(U_{i}|\eta ,y) & =D^{\frac{1}{2}}z_{i}^{\prime }{\left(\sigma^{2}I_{n_{i}}+z_{i}Dz_{i}^{\prime }\right)}^{-1}\left(y_{i}-x_{i}^{\prime }\beta \right),\\ Var(U_{i}|\eta ,y) & =I_{q}-D^{\frac{1}{2}}z_{i}^{\prime }{\left(\sigma^{2}I_{n_{i}}+z_{i}Dz_{i}^{\prime }\right)}^{-1}z_{i}D^{\frac{1}{2}}, \end{array}$$

i=1,…,m. We denote the conditional density of *U* as p(u|η,y) hereinafter.

### 2.2. Gibbs Sampler and the Final Fiducial Distribution

With both $r_{y}\left(\eta |u\right)$ and p(u|η,y) available, the fiducial distribution could be realized by a Gibbs sampler. Let $K\left(\eta ,\tilde{\eta }\right)\overset{▵}{=}\int r_{y}\left(\eta |u\right)p\left(u|\tilde{\eta },y\right)du$. By Equations (5) and (6), we have

(7) $$\begin{array}{cc} K(\eta ,\tilde{\eta }) & =\int C\left(X_{u},y\right)\frac{p(y|u,\eta )}{\sigma^{2}}p\left(u|y,\tilde{\eta }\right)du\\ \; & =\int C\left(X_{u},y\right)\frac{p(y,u|\eta )}{\sigma^{2}p\left(u\right)}p\left(u|y,\tilde{\eta }\right)du\\ \; & =\frac{p(y|\eta )}{\sigma^{2}}\int \frac{C(X_{u},y)}{p(u)}p\left(u|y,\eta \right)p\left(u|y,\tilde{\eta }\right)du\\ \; & \overset{▵}{=}\frac{p(y|\eta )}{\sigma^{2}}h\left(\eta ,\tilde{\eta }\right), \end{array}$$

where $h\left(\eta ,\tilde{\eta }\right)=\int \frac{C(X_{u},y)}{p(u)}p\left(u|y,\eta \right)p\left(u|y,\tilde{\eta }\right)du$ and p(y|η) is the likelihood function with *U* integrated out. Obviously, h(·,·) is symmetric and $\frac{p(y|\eta )}{\sigma^{2}}$ satisfies the detailed balance below:

(8) $$\frac{p(y|\tilde{\eta })}{{\tilde{\sigma }}^{2}}K\left(\eta ,\tilde{\eta }\right)=\frac{p(y|\tilde{\eta })}{{\tilde{\sigma }}^{2}}\frac{p(y|\eta )}{\sigma^{2}}h\left(\eta ,\tilde{\eta }\right)=\frac{p(y|\eta )}{\sigma^{2}}\frac{p(y|\tilde{\eta })}{{\tilde{\sigma }}^{2}}h\left(\tilde{\eta },\eta \right)=\frac{p(y|\eta )}{\sigma^{2}}K\left(\tilde{\eta },\eta \right).$$

The final fiducial density, as the stationary distribution of η, can be derived by the reversibility [20] of the Markov chain on η:

(9) $$r_{y}\left(\eta \right)\propto \frac{p(y|\eta )}{\sigma^{2}},$$

which is equivalent to a Bayesian LME with uniform prior on β and δ and prior $\frac{1}{\sigma }$ on σ.

In practice, once a *u* sampled by Equation (6) makes $X_{u}{\left(X_{u}^{\prime }X_{u}\right)}^{-1}X_{u}^{\prime }y$ close to *y*, the $Q_{y}\left(\sigma^{2}|u\right)$ in Equation (3) also becomes close to 0, making $Q_{y}\left(\theta |u\right)$ in Equation (4) nearly constant and the Monte Carlo Markov Chain (MCMC) degenerated. So, we restrict σ’s parameter space away from zero by a small $c_{0}>0$ so that $\sigma^{2}\in \left[c_{0},+\infty \right)$ and use the constrained fiducial quantity by a trimmed $\chi_{n-p-q}^{2}$ as follows:

(10) $$Q_{y}^{*}\left(\sigma^{2}|u\right)=\frac{y^{\prime }\left(I-X_{u}{\left(X_{u}^{\prime }X_{u}\right)}^{-1}X_{u}^{\prime }\right)y}{F_{\chi_{n-p-q}^{2}}^{-1}\left(F_{\chi_{n-p-q}^{2}}\left(\frac{y^{\prime }\left(I-X_{u}{\left(X_{u}^{\prime }X_{u}\right)}^{-1}X_{u}^{\prime }\right)y}{c_{0}}\right)U\left(0,1\right)\right)},$$

where F(·) is the cumulative distribution function, $F^{-1}\left(\cdot \right)$ is its inverse, and U(0,1) is a uniform random variable on (0,1). In this paper, we set $c_{0}=0.01$. The Gibbs sampling algorithm is given in Algorithm 1.
**Algorithm 1:** Gibbs sampling for $r_{y}\left(\eta \right)$Initialize $U^{\left(0\right)}$ by $U_{i}^{\left(0\right)}\overset{i.i.d}{∼}N\left(0,I_{q}\right)$, i=1,⋯,m.Given current $U^{\left(l\right)}$, sample $\beta^{\left(l\right)}$, $\delta^{\left(l\right)}$ from Equation (4) and $\sigma^{\left(l\right)}$ from Equation (10), respectively.Update $U^{\left(l+1\right)}$ by Equation (6).For l=0,1,2,⋯ iterate between step 2 to 3 until the MCMC chain gets well mixed.

**Remark 1.**  *We can also start with Equation (9) by setting corresponding prior in ready-made Bayesian packages that employ algorithms with better quality like HMC or NUTS [21]. However, HMC is time consuming and loses the advantages of direct inference for $\sigma_{j}=0$. The histogram of a Gibbs sample on δ can directly reflect how much the distribution is concentrated around zero. So, we mainly use Gibbs in this study and leave HMC for future research.*

**Remark 2.**  *When D is non-diagonal, $U_{i}\overset{i.i.d}{∼}N\left(0,I_{q}\right)$ becomes $U_{i}\overset{i.i.d}{∼}N_{q}\left(0,R\right)$, where R is the correlation matrix. And p(u|η,y) becomes p(u|η,R,y). Given U=u, the fiducial density of R, say $r_{u}\left(R\right)$, is also available [22]. The Gibbs sampler turns into iterations among three parts: $r_{y}\left(\eta |u\right)$, p(u|η,R,y) and $r_{u}\left(R\right)$. The fiducial density here is no longer equivalent to Equation (9) and cannot be readily implemented by Bayesian packages. We also leave this for future research.*

## 3. Fiducial Inference for LME

Given the observed *y*, the fiducial distribution of a parameter of interest g=g(η) can be given as

$$F_{y}\left(g\right)=\int_{\left\{g(\eta )\leq g\right\}}r_{y}\left(\eta \right)d\eta .$$

### 3.1. Interval Estimation

A 1−α fiducial interval for parameter g(η) is formed by the $\frac{\alpha }{2}$ and $1-\frac{\alpha }{2}$ quantiles of $F_{y}\left(g\right)$, denoted as $g_{\frac{\alpha }{2}}$ and $g_{1-\frac{\alpha }{2}}$, respectively, which are the solutions of

$$F_{y}\left(g_{\frac{\alpha }{2}}\right)=\frac{\alpha }{2},F_{y}\left(g_{1-\frac{\alpha }{2}}\right)=1-\frac{\alpha }{2}.$$

Then, the 1−α equal-tailed fiducial interval is $\left[g_{\frac{\alpha }{2}},g_{1-\frac{\alpha }{2}}\right]$.

Once we find a numerical sample of η as Section 2.2 described, the 1−α fiducial interval of g=g(η) can be obtained from the empirical distribution of $F_{y}\left(g\right)$, which is given in Algorithm 2.
**Algorithm 2:** Interval estimation for g(η)Generate $\eta_{1},\ldots ,\eta_{N}$ according to Algorithm 1.Compute $g_{i}=g\left(\eta_{i}\right),i=1,\cdots ,N$, and sort $g_{i}$ in ascending order to get $\left(g_{\left(1\right)},\ldots ,g_{\left(N\right)}\right)$.Find the $\frac{\alpha }{2}$ sample quantile $g_{\left(\left[N\cdot \frac{\alpha }{2}\right]\right)}$ and $1-\frac{\alpha }{2}$ sample quantile $g_{(\left[N\cdot \left(1-\frac{\alpha }{2})\right]\right)}$. Then $\left[g_{\left(\left[N\cdot \frac{\alpha }{2}\right]\right)},g_{(\left[N\cdot \left(1-\frac{\alpha }{2})\right]\right)}\right]$ is the 1−α fiducial interval estimation.

Noting that $\sigma_{j}$ is conventionally nonnegative, we use its zero-truncated distribution to construct the CI such that the left end is $\max\{0,\sigma_{(N\frac{\alpha }{2}),j}\}$. According to remark 2.1 and theorem 2.2 in [17], a truncated fiducial quantity uniquely solved from the fiducial structural equations has the same theoretical property as the untruncated one for inference in a restricted parameter space.

### 3.2. Fiducial p-Value

For a hypothesis test $H_{0}$:$g\left(\eta \right)=g_{0}$ with a two-sided alternative, the fiducial *p*-value is

$$p=2min\{F_{y}\left(g_{0}\right),1-F_{y}\left(g_{0}\right)\}.$$

Given a significance level α, $H_{0}$ is rejected when p<α. The computing procedure is given in Algorithm 3.
**Algorithm 3:** Fiducial *p*-value for $H_{0}$:$g\left(\eta \right)=g_{0}$Generate $\eta_{1},\ldots ,\eta_{N}$ according to Algorithm 1.Compute $g_{i}=g\left(\eta_{i}\right),i=1,\cdots ,N$.Calculate $p=2\min\left\{\frac{\sum_{i=1}^{N}1_{\left[g_{i}>g_{0}\right]}}{N},\frac{\sum_{i=1}^{N}1_{\left[g_{i}\leq g_{0}\right]}}{N}\right\}$.Reject $H_{0}$ if p<α.

**Remark 3.**  *Algorithms 2 and 3 are routine constructions of the CI and p value in unimodal distributions. All the histograms of δ that we have plotted so far are unimodal in a Gibbs sampler, probably because the sign of δ and that of U are interdependent in model (2) while Gibbs updates δ largely based on U. But, this does not mean the bimodal shape will not occur for δ in other algorithms bypassing the sampling of r(θ|u,y) since δ in Equation (9) is symmetric at about 0. For a bimodal distribution, routine p value and CI are not applicable but a histogram or density curve can directly tell.*

## 4. Simulation

### 4.1. Confidence Intervals

We compare the coverages and lengths of CIs constructed by our method (Fiducial), the profiling method (Profiling) in R package lme4 [9], the Bayesian LME (Bayeisan) with default prior in R package brms (version 2.22.0) [10], and the nonparametric bootstrap (Bootstrap) in [13]. We generate a chain over 6000 in length, with the first 300 as a warmup, and select every 3 steps to gather a sample size N = 2000 for the Gibbs sampler in Algorithm 1. We also try the HMC in Remark 1 by setting a uniform prior for β and $\sigma_{j}$, and $\frac{1}{\sigma }$ is approximated by an inverse gamma prior IG(0.001, 0.001) in brms (The density of IG(a, b) is $\frac{b^{a}e^{-b/\sigma }}{\Gamma \left(a\right)\sigma^{a+1}}$, σ>0. $\frac{1}{\sigma }=\lim_{b\to 0}\lim_{a\to 0}\frac{b^{a}e^{-b/\sigma }}{\Gamma \left(a\right)\sigma^{a+1}}$ thus can be approximated by IG(a,b) with a and b close to 0.) Bootstrap CIs are constructed by percentiles of 2000 replicates resampled among subjects, and each replicate is estimated by REML (lmer in package lme4). All MCMCs are set as a single chain with a sample size of 2000. Coverages (nominal level 95%) are annotated in figures and tables, averaged over 1000 runs.

**Figure 1:**
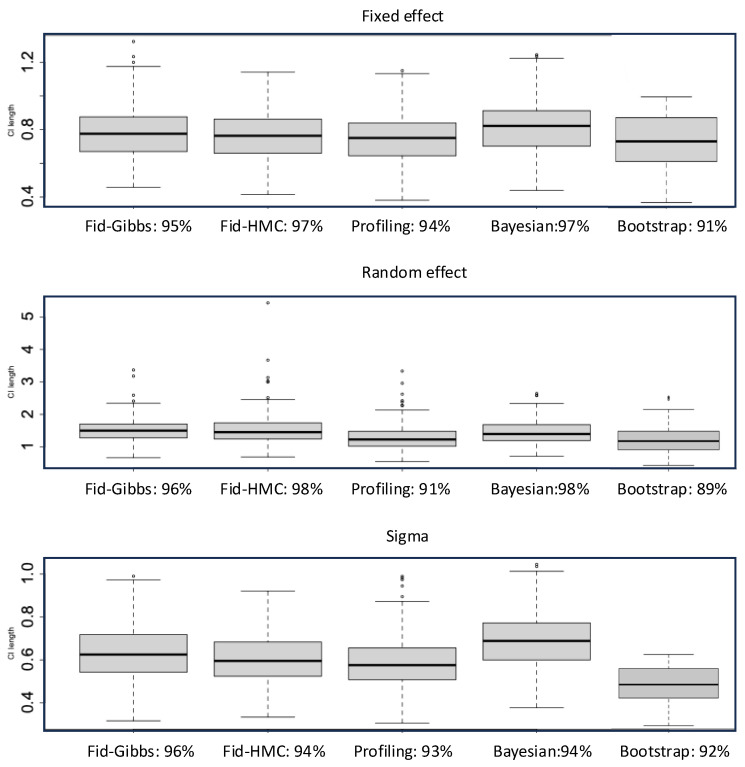
Confidence intervals in example 1.

**Example 1.**  *$y_{ij}=\beta_{0}+z_{ij}\gamma_{i1}+e_{ij}$ with $z_{ij}∼N\left(0,1\right)$, $\gamma_{i1}∼N\left(0,\sigma_{1}^{2}\right)$, $e_{ij}∼N\left(0,\sigma^{2}\right)$, 1≤j≤2, 1≤i≤10, $\beta_{0}=0.8$, $\sigma_{1}=0.75$, σ=0.7. In general, the Profiling CIs are narrower but susceptible to low coverages (Figure 1). Fiducial CIs are comparable to the Bayesian except those on $\sigma_{1}$ are often wider. This can be improved by HMC yet it is much slower. We use Gibbs for our method afterward. Bayesian LME is time-consuming yet performs well, partly due to its advantages in small samples and partly due to the suitability of the default prior for this parameter setting. Bootstrap has the lowest coverages; perhaps the sample size here is too small for it to take effect. The relative CPU time for model fit plus CI construction in fiducial (Gibbs), profiling, Bayesian, and bootstrap is about 2:1:30:40. Given the underperformance of bootstrap in coverage and computation efficiency, we do not discuss it in later examples. The variations in CI length are similar across different methods. So, we summarize the median length in the table hereinafter.*

**Example 2.**  *$y_{ij}=\beta_{0}+\gamma_{i0}+z_{ij}\gamma_{i1}+e_{ij}$ with $\gamma_{i0}∼N\left(0,\sigma_{0}^{2}\right)$, $\sigma_{0}=0$, and the others the same as example 1. That is, we add a false random effect $\gamma_{i0}\equiv 0$. Bayesian LME is unable to directly detect zero variance (coverage = 0 for $\sigma_{0}$, Table 1). Profiling CIs can have zero end points to keep reasonable coverages, but other parameters are affected, especially σ. Also, 5%∼10% of lmer’s output contains NA or ∞ when $n_{i}=q=2$. This is not a big issue when $n_{i}=4>q$, but σ’s coverage is still lower. In contrast, fiducial CIs capture both zero and nonzero variances in random effects and have stable coverages (Table 1).*

**Example 3.**  *$y_{i}=\beta_{0}+\gamma_{i0}+i\cdot \beta_{1}+i\cdot \gamma_{i1}+e_{i}$ with $\gamma_{i0}$, $\gamma_{i1}$, $e_{i}$ all from N(0,1), i=1,...,10, i.e., a longitudinal setting with $n_{i}=1$, q=2 so $n_{i}<q$. lmer does not work in this scenario, which makes profiling and bootstrap inapplicable. Bayesian CIs, overall, are wider with lower coverages than fiducial CIs (Table 2).*

### 4.2. Zero-Variance Test for Random Effects

**Figure 2:**
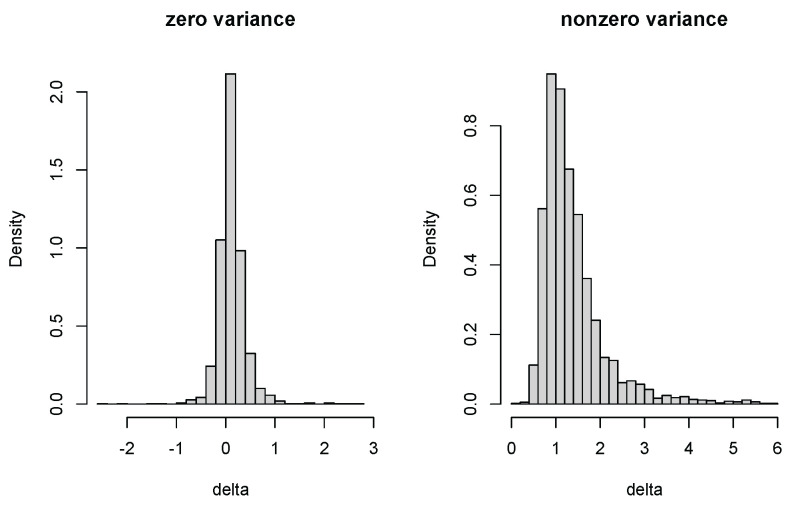
Fiducial density for δ=0 and δ=1.

**Figure 3:**
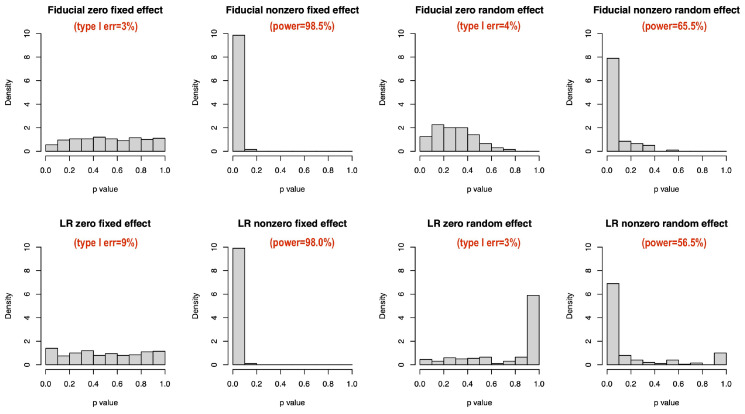
*p* value comparison for fixed and random effects, n = 20.

We plot the histograms of fiducial samples on zero and nonzero δ. They are indeed different (Figure 2). Negative δ generated by Equation (4) helps in measuring the significance of the random effect. We further compare *p* value distributions under null (=0) and alternative (≠0) hypotheses by treating example 2 as $y_{ij}=\beta_{0}+\gamma_{i0}+z_{ij}\beta_{1}+z_{ij}\gamma_{i1}+e_{ij}$ with $\beta_{1}=0$ and others unchanged. Frequentist *p* values are calculated by the package lmerTest [23] with fixed effects based on Satterthwaite-approximated t tests and random effects based on likelihood ratio (LR) tests. Fiducial *p* values are calculated simultaneously as described in Section 3. The type I error and power at a 5% significance level are annotated in Figure 3. The Satterthwaite *p* value has a higher type I error than the fiducial test (Figure 3). The type I errors of the fiducial and LR tests do not differ much, but the power of the fiducial test is higher. Moreover, the LR *p* value has a weird peak near 1 under alternative hypothesis (Figure 3), which is an obvious drawback in small data analysis. From this perspective, we think our fiducial approach can contribute to the inference in LME for small samples.

### 4.3. Comparison with GFIlmm

**Figure 4:**
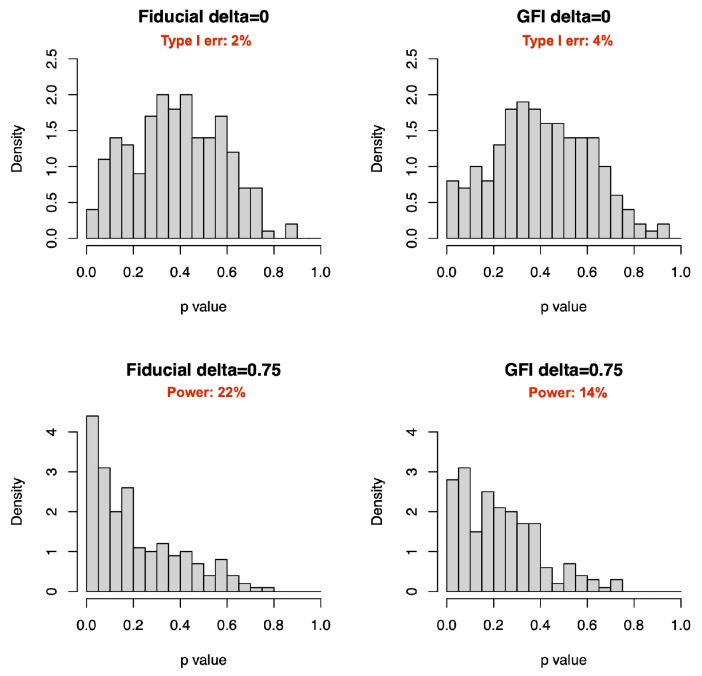
*p* value distributions on zero and nonzero random effects ($n_{i}=2$, m = 10).

For the GFIlmm in [19] (simplified as GFI hereinafter), we set a simpler scenario that their R package gfilmm (version 2.0.5) can run for comparsion: $y_{ij}=\beta_{0}+\gamma_{i}+\beta_{1}x_{ij}+e_{i}$. Here, $\beta_{0}=0.8$, $\beta_{1}=0.5$, $\gamma_{i}∼N\left(0,\delta^{2}\right)$, $x_{ij}∼N\left(0,1\right)$ and $e_{i}∼N\left(0,1\right)$, j = 1, 2; i=1,…,10. Intervals are added as $y_{ij}-0.01<y_{ij}<y_{ij}+0.01$. Performances on both zero and nonzero δ are compared (Table 3). The CIs in the nonzero case are comparable, and GFI is better in the zero case. We also plot histograms of the two fiducial *p* values. At a 5% significance level, our method has lower type I error and higher power than GFI (Figure 4).

## 5. Empirical Analysis

**Figure 5:**
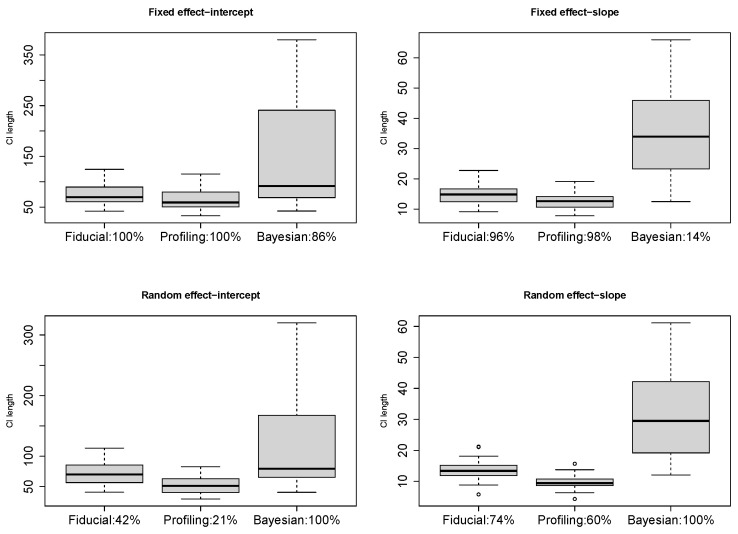
CI length in resampled sleep data.

We use the sleepstudy data in R package lme4 as an illustration, which contains a sleep-deprived group of 18 subjects for 10 days of the study in [24]. Day0 and Day1 were adaptation and training, so altogether 18×8=144 observations. The reaction time of the *i*th subject on $Day_{j}$ after sleep deprivation is modeled as

$$reaction_{ij}=\beta_{0}+\gamma_{i1}+\beta_{1}Day_{j}+\gamma_{i2}Day_{j}+e_{ij},$$

with $\gamma_{i1}∼N\left(0,\sigma_{1}^{2}\right)$, $\gamma_{i2}∼N\left(0,\sigma_{2}^{2}\right)$, and $e_{i}∼N\left(0,\sigma^{2}\right)$. Both the fixed and random effects are significant [9]. We resample 25% of the data for a subset with n=9×4=36 to see how much the inference based on a smaller sample agrees with that on the whole dataset. We conduct resampling 50 times and plot the CI lengths of $\beta_{0}$, $\beta_{1}$, $\sigma_{1}$, and $\sigma_{2}$ in Figure 5. Different from the simulation, the Bayesian intervals are much wider this time since the default prior does not well suit the real data. We annotate the percentage of CIs excluding 0 in Figure 5 as an estimated power, on which the fiducial LME are generally higher. We further try a combination of m=18 and $n_{i}=2$, retaining n = 36, but neither lmer nor brms can fit all the cases successfully this time. Our fiducial LME still works. The percentages of significance are 100% on $\beta_{0}$, 80% on $\beta_{1}$, 21% on $\sigma_{1}$, and 74% on $\sigma_{2}$, respectively.

## 6. Conclusions

We study the fiducial inference in LME with the group-level variation innovatively modeled as coefficients. We derive the fiducial density by the stationary theory of Markov chains and demonstrate its advantages in small sample sizes by using simulation and real data. Our study facilitates the inference of zero variance in random effects and reveals a deep relationship between fiducial and Bayesian inference. On one hand, it confirms the rationality of the commonly used noninformative prior in Bayesian LME. On the other, the equivalence to a Bayesian LME is only for independent random effects. It is difficult to make a direct inference of zero variance in random effects if we simply treat the final density as a posterior distribution with a flat prior. But, from the perspective of fiducial structural equations, it turns out to be feasible.

We compare four inferential methods on $n_{i}>q$, $n_{i}=q$, and $n_{i}<q$ respectively. Our approach outperforms others on $n_{i}\leq q$. This is helpful in prescreening random effects since *q* can easily exceed $n_{i}$ when we test if all the predictors have random effects. The minimal sample size on which our fiducial LME can work stably is much smaller than other methods. We also tried the parametric bootstrap in lme4. But, its improvement over nonparametric bootstrap is quite limited regarding coverage under the scenarios in Section 4.1.

There is also much space for improvement. The Gibbs algorithm is not fine enough. More advanced numerical algorithms can also be considered and will be studied in the future. The theoretical property of the truncated δ in the fiducial sample and the inference for correlated random effects (non-diagonal D) also need further investigation.

## Figures and Tables

**Table 1:** Coverage (length) of CIs in example 2.

$n_{i}=2,m=10$	$\beta_{0}=0.8$	$\sigma_{0}=0$	$\sigma_{1}=0.75$	σ=0.7
Fiducial	0.99 (0.99)	0.95 (1.01)	0.97 (1.63)	0.95 (0.71)
Profiling	0.93 (0.80)	0.97 (0.70)	0.89 (1.38)	0.87 (0.64)
Bayesian	0.97 (1.03)	0.00 (0.98)	0.97 (1.49)	0.96 (0.77)
$n_{i}=4,m=5$	$\beta_{0}=0.8$	$\sigma_{0}=0$	$\sigma_{1}=0.75$	σ=0.7
Fiducial	1.00 (1.30)	0.98 (1.50)	0.96 (2.21)	0.95 (0.60)
Profiling	0.95 (0.86)	0.99 (0.65)	0.93 (1.78)	0.92 (0.54)
Bayesian	1.00 (1.15)	0.00 (1.38)	0.97 (1.99)	0.95 (0.67)

**Table 2:** Coverage (length) of CIs in example 3.

$n_{i}=1$, m=10	$\beta_{0}=10$	$\beta_{1}=0$	$\sigma_{0}=1$	$\sigma_{1}=1$	σ=1
Fiducial	0.99 (16.31)	0.98 (3.36)	1.00 (9.89)	0.98 (2.06)	1.00 (6.26)
Profiling	NA	NA	NA	NA	NA
Baysian	0.97 (20.87)	0.94 (4.36)	0.97 (9.01)	0.96 (2.79)	0.77 (8.58)

**Table 3:** Coverages (length) of CIs in comparison with GFI.

Parameter	$\beta_{0}=0.8$	$\beta_{1}=0.5$	δ=0.75	σ=1
Fiducial	0.97 (1.56)	0.94 (0.83)	0.98 (1.55)	0.96 (0.82)
Profling	0.94 (1.43)	0.92 (0.82)	0.94 (1.25)	0.96 (0.79)
GFI	0.96 (1.60)	96 (0.93)	1.0 (1.45)	0.95 (0.93)
Parameter	$\beta_{0}=0.8$	$\beta_{1}=0.5$	δ=0	σ=1
Fiducial	0.98 (1.23)	0.95 (1.00)	0.98 (1.21)	0.95 (0.78)
Profling	0.93 (0.96)	0.93 (0.75)	0.96 (0.88)	0.91 (0.66)
GFI	0.97 (1.15)	0.94 (0.82)	0.98 (1.09)	0.96 (0.77)

## Data Availability

No new data were created or analyzed in this study.

## References

[1] Jiang J. (2007). Linear and Generalized Linear Mixed Models and Their Applications.

[2] Buscemi S., Plaia A. (2019). Model selection in linear mixed-effect models. Asta-Adv. Stat. Anal..

[3] Zhang X., Zou G., Liang H. (2014). Model averaging and weight choice in linear mixed-effects models. Biometrika.

[4] Waterman M.J., Birch J.B., Abdel-Salam A.-S.G. (2015). Several nonparametric and semiparametric approaches to linear mixed model regression. J. Stat. Comput. Simul..

[5] Dion C. (2014). New adaptive strategies for nonparametric estimation in linear mixed models. J. Stat. Plan. Inference.

[6] Jiang J. (2017). Asymptotic Analysis of Mixed Effects Models: Theory, Applications, and Open Problems.

[7] Breslow N.E., Clayton D.G. (1993). Approximate inference in generalized linear mixed models. J. Am. Stat. Assoc..

[8] Chung Y., Rabe-Hesketh S., Dorie V., Gelman A., Liu J. (2013). A non-degenerate estimator for variance parameters in multilevel models via penalized likelihood estimation. Psychometrika.

[9] Bates D. (2014). Fitting linear mixed-effects models using lme4. arXiv.

[10] Burkner P.-C. (2017). brms: An r package for bayesian multilevel models using stan. J. Stat. Softw..

[11] Das S., Krishen A. (1999). Some bootstrap methods in nonlinear mixed-effect models. J. Stat. Plan. Inference.

[12] Flores-Agreda D., Cantoni E. (2019). Bootstrap estimation of uncertainty in prediction for generalized linear mixed models. Comput. Stat. Data Anal..

[13] Thai H.-T., Mentre F., Holford N., Veyrat-Follet C., Comets E. (2013). A comparison of bootstrap approaches for estimating uncertainty of parameters in linear mixed-effects models. Pharm. Stat..

[14] Fisher R.A. (1937). On a point raised by m. s. bartlett on fiducial probability. Ann. Hum. Genet..

[15] Hannig J., Iyer H., Lai R.C.S., Lee T.C.M. (2016). Generalized fiducial inference: A review and new results. J. Am. Stat. Assoc..

[16] Martin R., Liu C. (2013). Inferential models: A framework for prior-free posterior probabilistic inference. J. Am. Stat. Assoc..

[17] Li X., Su H., Liang H. (2018). Fiducial generalized p-values for testing zero-variance components in linear mixed-effects models. Sci. China Math..

[18] Lidong E., Hannig J., Iyer H.K. (2008). Fiducial intervals for variance components in an unbalanced two-component normal mixed linear model. J. Am. Stat. Assoc..

[19] Cisewski J., Hannig J. (2012). Generalized fiducial inference for normal linear mixed models. Ann. Stat..

[20] Grimmett G.R., Stirzaker D.R. (2001). Probability and Random Processes.

[21] Betancourt M. (2017). A Conceptual Introduction to Hamiltonian Monte Carlo. arXiv.

[22] Shi W.J., Hannig J., Lai R.C.S., Lee T.C.M. (2021). Covariance estimation via fiducial inference. Stat. Theory Relat. Fields.

[23] Kuznetsova A., Brockhoff P.B., Christensen R.H.B. (2017). lmerTest package: Tests in linear mixed effects models. J. Stat. Softw..

[24] Belenky G., Wesensten N., Thorne D., Thomas M., Sing H., Redmond D., Russo M., Balkin T. (2003). Patterns of performance degradation and restoration during sleep restriction and subsequent recovery: A sleep dose-response study. J. Sleep Res..

